# New York Letter

**Published:** 1902-08

**Authors:** 


### Domestic Correspondence.

### NEW YORK LETTER.

To the Editor:

Sir,—Notwithstanding the heated term, a goodly number met at Dr. C. D. Cook's office on June 3, which was to be the last dental meeting of The New York Institute of Stomatology until the autumn. Several interesting papers were read and discussed. Dr. D. A. Fuller gave a brief synopsis of an interesting case in practice, which, briefly speaking, was in relation to the advisability and beneficial results in the use of quinine in indicated instances.

Dr. W. M. Whitlock described his manner of opening and cleansing root-canals, which was surely lucidly explained and clearly demonstrated by the aid of different instruments used for that purpose. It was the general opinion of the many gentlemen present that Dr. Whitlock's method was certainly a scientific one.

Dr. J. Morgan Howe, the honored president of the society, read another interesting paper, entitled "Prevention of Decay." A paper of this import and value must be carefully read and mentally digested to get the real essence of it; suffice it now to say that Dr. Howe's excellent paper, while conservative, uttered warning notes to those advocating "extension for prevention," but rather recommended that our efforts be directed in the line of saving tooth-structure by thorough prophylaxis and conservative methods in filling, instead of wholesale trimming of tooth-structure to attempt to prevent future decay.

After the interesting discussion following the paper, the meeting adjourned until autumn, after partaking of a collation, which was enjoyed through the genial hospitality of the host.

"Lochinvar."

